# Hot Deformation Behavior and Processing Maps of Fe-30Mn-0.11C Steel

**DOI:** 10.3390/ma11101940

**Published:** 2018-10-11

**Authors:** Jianmei Kang, Yuhui Wang, Zhimeng Wang, Yiming Zhao, Yan Peng, Tiansheng Wang

**Affiliations:** 1 State Key Laboratory of Metastable Materials Science and Technology, Yanshan University, Qinhuangdao 066004, China; 15076079693@163.com (J.K.); 15036562990@163.com (Y.Z.); tswang@ysu.edu.cn (T.W.); 2 National Engineering Research Center for Equipment and Technology of Cold Strip Rolling, Yanshan University, Qinhuangdao 066004, China; Zhimeng_ng_wang12@163.com (Z.W.); pengyan@ysu.edu.cn (Y.P.); 3 College of Engineering, Yantai Nanshan University, Yantai 265713, China

**Keywords:** Fe-30Mn-0.11C steel, constitutive equation, processing maps, recrystallization diagrams

## Abstract

Hot deformation behavior of Fe-30Mn-0.11C steel was investigated. Hot compression tests were carried out at various temperatures ranging from 800 °C to 1200 °C and at different strain rates of 0.01 s^−1^ to 10 s^−1^. The constitutive equation based on peak stress was established. Hot processing maps at different strains and recrystallization diagrams were also established and analyzed. The results show that dynamic recrystallization easily occur at high deformation temperatures and low strain rates. Safe and unstable zones are determined at the true strain of 0.6 and 0.7, and the hot deformation process parameters of partial dynamic recrystallization of the tested steel are also obtained.

## 1. Introduction

Hot processing is an important metal forming process, and a combination of work hardening, dynamic recovery, and dynamic recrystallization processes [1,2]. The occurrence of dynamic recrystallization, which can refine grains and reduce deformation resistance, significantly affects the forming properties, microstructure, and mechanical properties of the materials [3]. The flow stress model can reflect changes in the microstructure of the material and can be utilized to predict the hot deformation behavior of materials [4]. A hot processing map based on a dynamic model reflects a hot deformation mechanism of the material and is a powerful tool for the design and optimization of metal processing [5]. To study hot deformation behavior of materials, a flow stress model is established and a hot processing map is analyzed.

The Fe-Mn-C alloy system has been of interest for the development of materials capable of work-hardening to obtain very high strength levels [6,7]. The alloy system is capable of exhibiting dislocation accumulation, solid–solution hardening, twinning, and strain-induced martensitic transformations, depending on the composition and deformation temperature [8,9,10]. As a potential low temperature steel [11], numerous studies on microstructure, deformation products and mechanical behavior of low temperature Fe-30Mn steels can be found, in particular, the microstructure refinement and the effect of alloy elements was studied [12,13]. In contrast, only a few studies on hot deformation behavior are available [14,15].

In the current work, the hot deformation behavior of the Fe-30Mn-0.11C steel including determination of hot deformation equations and hot processing maps, analyzing austenite recrystallization rule, and finding recrystallization region maps was studied. It provides a theoretical guidance for the industrial production of the studied alloy using hot processing.

## 2. Experimental Procedure

### 2.1. Sample Preparation and Hot-Compression Tests

**Figure 1:**
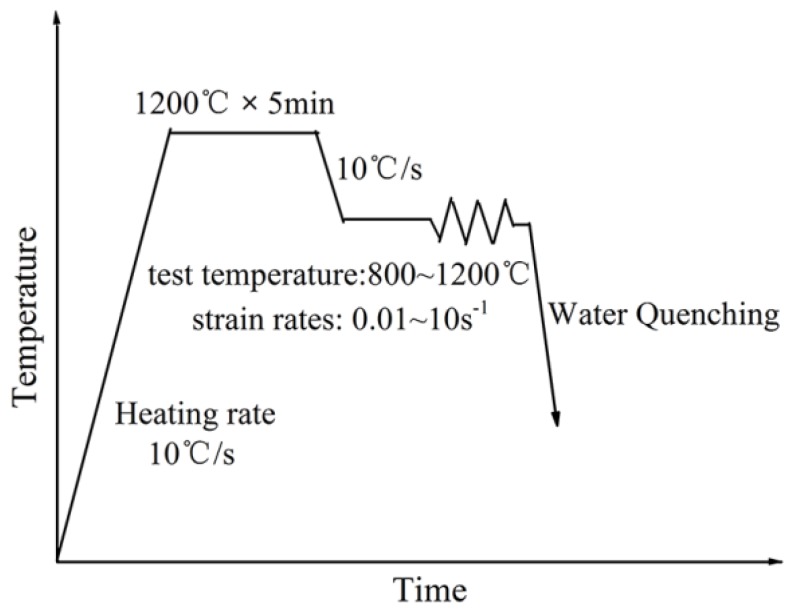
A schematic representation of hot compression process.

A high-Mn austenitic steel containing 0.11C, 30.5Mn (in wt %), which was coming from an experimental cast was selected for the present study where an ingot was produced from a vacuum induction furnace and subsequently forged in the temperature range of 800–1100 °C to form a bar with a diameter of 35 mm. Cylindrical samples with a diameter of 10 mm and height of 15 mm were cut from the hot-forged bar. A single pass hot compression test was carried out in a Gleeble 3800 thermo-mechanical simulator (Dynamic Systems Inc, Austin, TX, USA). The tantalum sheets coated with high-temperature lubricant were attached to both ends of a sample before a single-pass hot compression test, which ensured homogeneous deformation of the specimen and preventing the specimen from sticking to the indenter at high temperatures. A thermocouple was welded at the mid-height position of the cylindrical specimen for precise temperature control and measurement. The schematic representation of hot compression process is shown in Figure 1. The specimens were heated from room temperature to 1200 °C at a heating rate of 10 °C/s and held for 5 min for homogenization, and then cooled to the different deformation temperature at a cooling rate of 10 °C/s. Hot compression was carried out using a 50% reduction in height (an equivalent true strain of 0.69) at various temperatures (800, 900, 1000, 1100 and 1200 °C) and different strain rates (0.01, 0.1, 1, and 10 s^−1^). During deformation, displacement and load were recorded for obtaining stress–strain curves. The specimens were water-quenched immediately after the deformation to preserve the high temperature microstructure.

### 2.2. Microstructural Observations

Microstructures of the starting material and hot compressed specimens were characterized using a light microscope (Zeiss, Jena, Germany). For the investigation, cylindrical hot-compressed specimens were vertically sectioned through the center parallel to the compression direction, and a quarter on the section was observed. The sections were mechanically polished and etched using 5 vol % nital solution.

## 3. Results and Discussion

### 3.1. Starting Material

**Figure 2:**
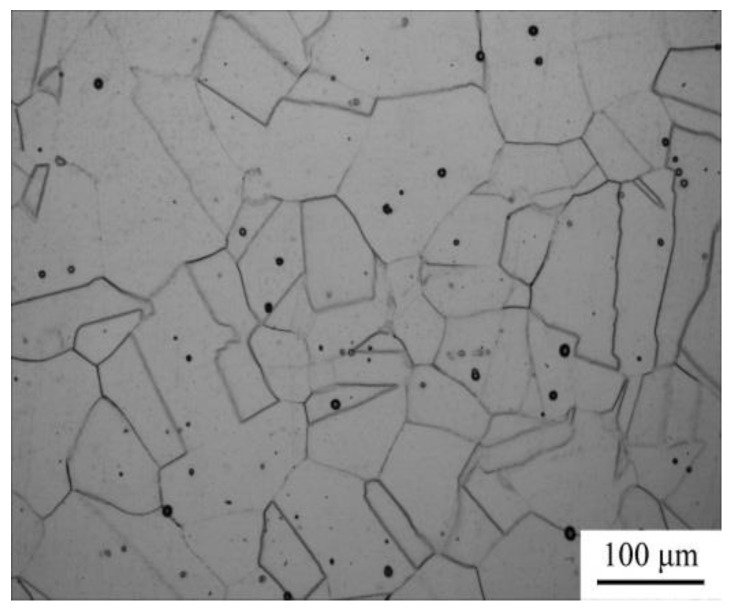
Microstructure of the starting material of Fe-30Mn-0.11C steel for hot compression tests.

A hot-forged high-Mn austenitic steel prior to hot compression (Figure 2) was used as the starting material. Figure 2 shows the microstructure of Fe-30Mn-0.11C steel with austenite grain size of 106 μm, where the average grain size was calculated using a linear intercept method.

### 3.2. True Stress–Strain Curves

**Figure 3:**
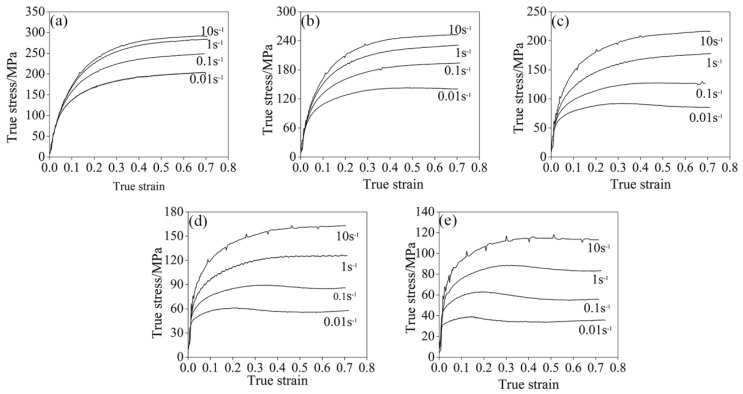
True stress–true strain curves of Fe-30Mn-0.11C steel at different temperatures and strain rates: (**a**) 800 °C; (**b**) 900 °C; (**c**) 1000 °C; (**d**) 1100 °C and (**e**) 1200 °C.

The true stress–strain curves at different deformation temperatures and various strain rates are displayed in Figure 3. The flow stress decreased with increasing deformation temperature. At a given deformation temperature, the flow stress increased with increasing the strain rate. The characteristics of flow curves included a peak stress followed by a flow-softening regime that could be recognized as dynamic recrystallization (DRX) [16]. This will be examined in detail later. The stress–strain curves indicated that a high deformation temperature and low strain rate were beneficial to dynamic recrystallization.

### 3.3. Hot Deformation Parameters

#### 3.3.1. Hot Deformation Constitutive Equation

Flow stress (σ) is affected by the deformation strain rate ($\dot{\varepsilon }$), temperature (*T*), and strain (*ε*) during hot deformation. The relationship between these parameters can be expressed using an appropriate constitutive equation. Flow stress analysis was carried out using a widely accepted Arrhenius type hyperbolic-sine relationship proposed by Sellars and Tegart [17,18]. There are three different equations: Equations (1)–(3) are applicable to low stress levels (*ασ* < 0.8), high stress levels (*ασ* > 1.2), and all stress levels, respectively:

(1) $$\;Z=\dot{\varepsilon }{\exp(Q/RT)=A}_{1}\sigma^{n_{1}}\;$$

(2) $$\;Z=\dot{\varepsilon }{\exp(Q/RT)=A}_{2}\exp(-\beta \sigma )\;$$

(3) $$\;Z=\dot{\varepsilon }{\exp(Q/RT)=A[\sin h(\alpha \sigma )]}^{n}\;$$

where the Zenner–Hollomon parameter (*Z*) is the temperature-compensated strain rate factor [19]; $\dot{\varepsilon }$ represents the strain rate (s^−1^); *A* denotes the structural factor, *α* means the stress level parameter; *n* is stress index; *β* is a constant; *Q* is the apparent activation energy for hot deformation (kJ/mol); *R* represents the gas constant (8.314 J/mol·K); and *σ* could be peak stress ($\sigma_{p}$), steady-state stress ($\sigma_{ss}$), or flow stress corresponding to a specified strain.

**Figure 4:**
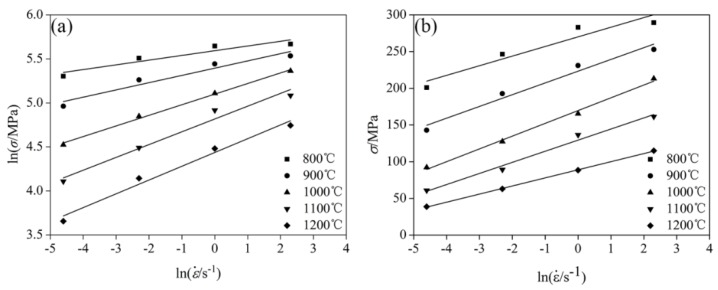
Relationship between peak stress and strain at different temperatures: (**a**) $\ln\dot{\varepsilon }-\ln\sigma$, and (**b**) $\ln\dot{\varepsilon }-\sigma$.

By taking the log function on both sides of the Equations (1) and (2) and partial differentiation at the constant temperature of *T*, the following Equations (4)–(6) are obtained:

(4) $$n_{1}={\left[\frac{\partial \ln\dot{\varepsilon }}{\partial \ln\sigma }\right]}_{T}$$

(5) $$\beta ={\left[\frac{\partial \ln\dot{\varepsilon }}{\partial \sigma }\right]}_{T}$$

(6) $$\;{\alpha =\beta /n}_{1}\;$$

ln*ε* versus ln*σ* and ln*ε* versus *σ* are respectively plotted in Figure 4. The value of *n*_1_ (*n*_1_ = 6.390185) and *β* (*β* = 0.064849) could be derived from the linear fitting of $\ln\dot{\varepsilon }$ versus lnσ and $\ln\dot{\varepsilon }$ versus σ. Furthermore, the value of the *α* (*α* = 0.010148) parameter could be obtained from Equation (6).

**Figure 5:**
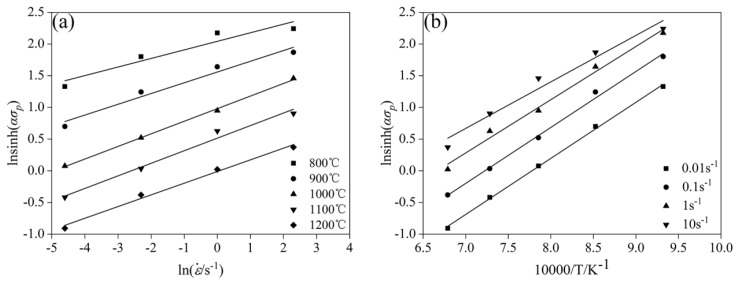
Relationship between peak stress and true strain and temperature: (**a**) ${\mathrm{lnsin}h(\alpha \sigma }_{p}-\ln\dot{\varepsilon }$, and (**b**) ${\mathrm{lnsin}h(\alpha \sigma }_{p})\;$− 1/*T.*

By taking the log function on both sides of Equation (3), the following Equations (7) and (8) are obtained and the following curves of $\ln\left[\sin h\left(\alpha \sigma_{p}\right)\right]$− $\ln\dot{\varepsilon }$ and $\ln\left[\sin h\left(\alpha \sigma_{p}\right)\right]$−1/*T* were respectively plotted in Figure 5.

(7) $$\ln\dot{\varepsilon }+Q/RT=\ln A+n\ln[\sin h(\alpha \sigma )]$$

(8)  lnZ=lnA+nln[sinh(ασ)] 

**Figure 6:**
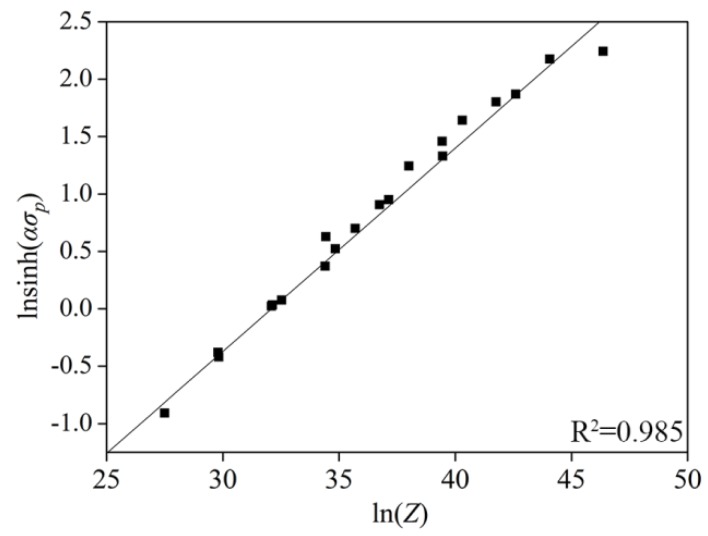
Schematic of $\ln\left[\sin h\left({\alpha \sigma }_{p}\right)\right]$ versus  lnZ.

The values of *n*, *Q*, and *A* could be derived from linear fitting using $\ln\left[\sin h\left(\alpha \sigma_{p}\right)\right]$ versus $\ln\dot{\varepsilon }$ and $\ln\left[\sin h\left(\alpha \sigma_{p}\right)\right]$ vs. 1/*T*, which is shown in Figure 6 (*n* = 5.645, *Q* = 393 kJ/mol, and $\ln A\;=\;8.698\;\times \;10^{13}$). The value of *Q* was slightly higher than that of the high entropy alloy [20] and similar to the Fe-20Mn-3Si-3Al steel [21]. Figure 6 indicates a linear relationship of the flow stress against the Zener–Hollomon parameter (*Z*). Therefore, the constitutive Equation (9) at *σ* = *σ_p_* could be obtained as:

(9) $$\dot{\varepsilon }=8.689\times 10^{13}\times {\left[\sin h(0.01015\sigma )\right]}^{5.645}\exp(-393,070/RT)$$

#### 3.3.2. Hot Deformation Characteristic Parameter Model

It is important to determine the hot deformation characteristic parameters and obtain its model [22]. Poliak and Jonas [23,24] believe that hot deformation is an irreversible process, and define critical stress as the stress in the appearance of additional thermodynamic degrees of freedom. The hot deformation characteristic parameters of the material are obtained from the relationship curve of work hardening rate, *θ(∂σ/∂ε)* and flow stress *σ*, and the method is called the P–J method. In this study, the characteristic parameter values at different deformation conditions are obtained based on the P–J method, and the model reflecting its relationship with *Z* parameters is established as follows Equation (10). Where $\sigma_{c}\;$ is critical stress, $\varepsilon_{c}$ is strain, $\sigma_{p}$ is peak stress, $\varepsilon_{p}$ is peak strain, $\sigma_{m}$ is maximum softening stress, $\varepsilon_{m}$ is strain, $\sigma_{ss}$ is steady-state stress, and $\varepsilon_{ss}$ is steady-state strain of materials for thermal deformation.

(10) $$\begin{array}{c} \;\sigma_{c}\;=\;{0.89\sigma }_{p}\\ \;\varepsilon_{c}\;=\;{0.4\varepsilon }_{p}\\ \;\sigma_{p}\;=\;{0.6273Z}^{0.1528}\\ \;\varepsilon_{p}\;=\;{0.00118Z}^{0.1717}\\ \;\varepsilon_{p}\;=\;{0.000927\dot{\varepsilon }}^{0.188}\exp(\frac{70,761}{RT})\\ \;\sigma_{c}\;=\;{1.4681Z}^{0.1236}\\ \;\varepsilon_{c}\;=\;{0.00504Z}^{0.102}\\ \;\sigma_{ss}\;=\;{0.2982Z}^{0.174}\\ \;\varepsilon_{ss}\;=\;{0.0489Z}^{0.0791}\\ \;\varepsilon_{m}\;=\;{0.00592Z}^{0.1321}\; \end{array}$$

#### 3.3.3. Dynamic Recrystallization Kinetic Model

The improved Avrami Equations (11) and (12) is used to describe dynamic recrystallization kinetics of materials [22]:

(11) $$\;X_{DRX}\;=\;1-\exp\left[-k{\left(\frac{\varepsilon -\varepsilon_{c}}{\varepsilon_{m}-\varepsilon_{c}}\right)}^{n}\right]\;$$

(12) $$\;X_{DRX}=\frac{\sigma_{recov}^{2}-\sigma^{2}}{\sigma_{s}^{2}-\sigma_{ss}^{2}}\;$$

where $X_{DRX}$ is the volume fraction of dynamic recrystallization, *k* and *n* are material Avrami constants, *σ_recov_* is the visual dynamic recover stress, and *σ_s_* is the saturation stress.

**Figure 7:**
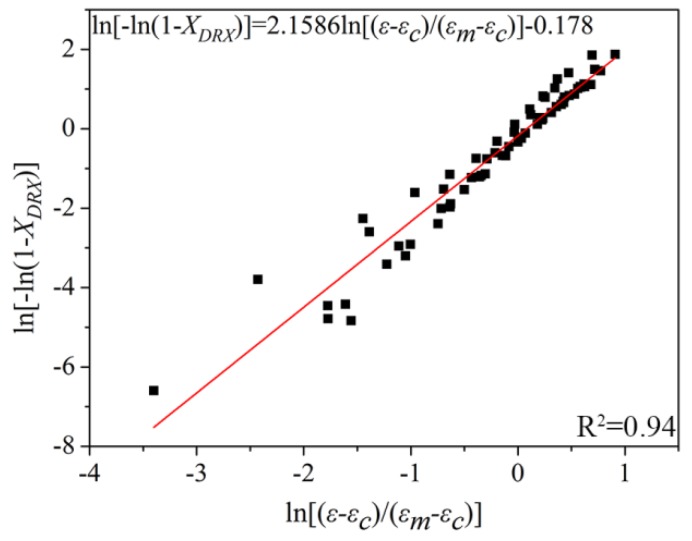
Schematic of $\ln\left[{-\ln(1-X}_{DRX})\right]$ versus $\ln\left(\frac{{\varepsilon -\varepsilon }_{c}}{\varepsilon_{m}{-\varepsilon }_{c}}\right)$ at different deformation conditions.

By taking the log function on both sides of Equation (11), the relationship curve of $\ln\left(-\ln\left(1-X_{DRX}\right)\right)$ versus −$\ln\left(\varepsilon -\varepsilon_{c}\right)/\left(\varepsilon_{m}-\varepsilon_{c}\right)$ is obtained, as shown in Figure 7.

The value of *k* and *n* could be derived from linear fitting of $\ln\left(-\ln\left(1-X_{DRX}\right)\right)$ versus $\ln\left(\varepsilon -\varepsilon_{c}\right)/\left(\varepsilon_{m}-\varepsilon_{c}\right)$ (*n* = 2.1586, *k* = 0.8369). Consequently, the dynamic recrystallization kinetic model could be obtained as:

(13) $$\;X_{DRX}\;=\;1-\exp\left[-0.8369{\left(\frac{\varepsilon -\varepsilon_{c}}{\varepsilon_{m}-\varepsilon_{c}}\right)}^{2.1586}\right]\;$$

**Figure 8:**
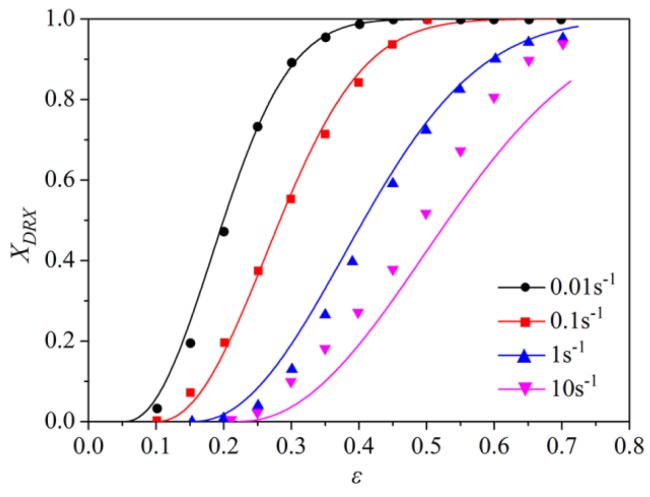
A comparison of calculated and measured values of the dynamic recrystallization percentage.

The obtained linear relationship (Equation (13)) makes it possible to predict *X_DRX_* at a temperature and strain rate. Figure 8 shows a comparison of the dynamic recrystallization percentage calculated using Equation (13) and measured values. The solid line represents the calculated value and the scattered point denotes the measured values. It can be observed that the measured values are very consistent with the calculated value.

#### 3.3.4. Microstructure Evolution Model

Since grain size largely determines the mechanical properties, such as hardness and toughness of the material, predicting product performance to establish the recrystallized grain size model is inevitable. The recrystallized grain size of materials at different deformation conditions is calculated using linear intercept method, and the relationship between grain size and *Z* parameters, and between deformation parameters, e.g., strain rate and deformation temperature are established and analyzed according to previous studies [25,26]. Thereafter, a model expressing *Z* parameter (Equation (14)) and a model expressing deformation temperature and strain rate (Equation (15)) are established:

(14) $$\;D_{DRX}\;=\;{77,365.79Z}^{-0.25}\;$$

(15) $$D_{DRX}\;=\;{3,592,759\dot{\varepsilon }}^{-0.12763}\exp(\frac{-141,071}{RT})$$

**Figure 9:**
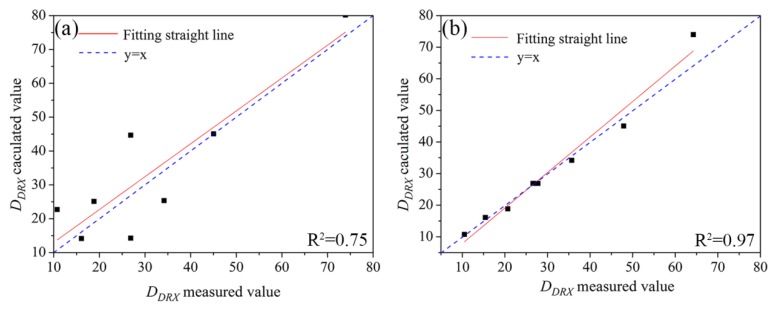
A comparison of measured value and calculated value of recrystallized grain size: (**a**) model represented by *Z* parameters, and (**b**) model expressed by strain rate and deformation temperature.

Figure 9 shows a comparison of the calculated and measured values obtained using above two recrystallized grain size models. It can be seen that the model with deformation temperature and strain rate has a higher calculation accuracy. A model expressed using the Z parameter (Equation (14)) displayed higher calculation error, which may be due to the *Z* parameter being an intermediate variable.

#### 3.3.5. Recrystallization Area Maps

**Figure 10:**
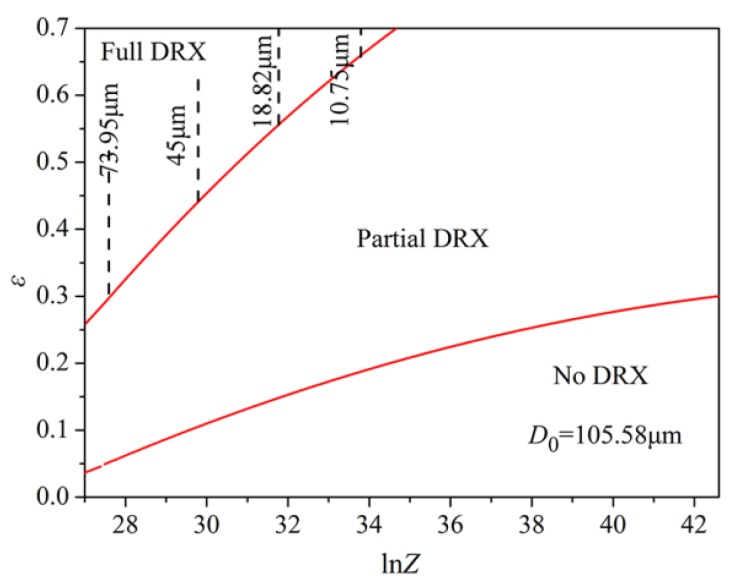
DRX map of the experimental steel.

In order to reveal the relationship between deformation temperature, strain rate, strain and recrystallization percentage, and recrystallized grain size more clearly and intuitively, a dynamic recrystallization area map of the material based on the dynamic recrystallization kinetics model and the recrystallized grain size model was established. The dynamic recrystallization area map (with *Z* parameters and strain values as variables) is shown in Figure 10. The *Z* parameter, which is a function of deformation temperature and strain rate, comprehensively indicates the influence of strain rate and deformation temperature on the mechanical behavior of hot deformation. Therefore, a recrystallization area map is an important tool for developing a reasonable hot processing process in industrial production.

It can be seen from Figure 10 that dynamic recrystallization varies with a changing *Z* parameter. The larger the *Z* parameter is, the less likely it is that dynamic recrystallization occurs. In the fully dynamic recrystallization zone, the larger the *Z* parameter is, the smaller the recrystallized grain size is. For instance, at the deformation temperature of 1000 °C and the strain rate of 0.01, the *Z* parameter had a minimum value in the fully dynamic recrystallization zone. The recrystallized grain size was 10.75 μm, which is the optimum hot deformation process parameter.

#### 3.3.6. Hot Processing Maps

The hot processing map based on the dynamic material model is a graph developed in the past two decades for studying the hot processing performance of materials and is an important tool for optimizing hot processing parameters of materials [27]. At a certain strain, the power dissipation efficiency and the instability parameter are superimposed in the form of contour lines on a two-dimensional plane composed of strain rate and temperature to create a hot processing map. The power dissipation efficiency and instability criteria are obtained using Equations (16) and (17), respectively:

(16) $$\;\eta =\frac{2m}{m+1}\;$$

(17) $$\xi (\dot{\varepsilon })=\frac{\partial \ln(\frac{m}{m+1})}{\partial \ln\dot{\varepsilon }}+m<0$$

where *η* is the energy dissipation factor, ξ($\dot{\varepsilon }$) is the instability parameter, and *m* is the strain rate sensitivity coefficient where$\;m\;=\;\partial (\ln\sigma )/\partial (\ln\dot{\varepsilon })$.

At a certain strain, the curves of *η* and ξ($\dot{\varepsilon }$) with respect to strain rate and deformation temperature were also obtained, and the power dissipation diagram and the rheological instability diagram were obtained. A hot working diagram of the studied steel at this strain was then obtained.

**Figure 11:**
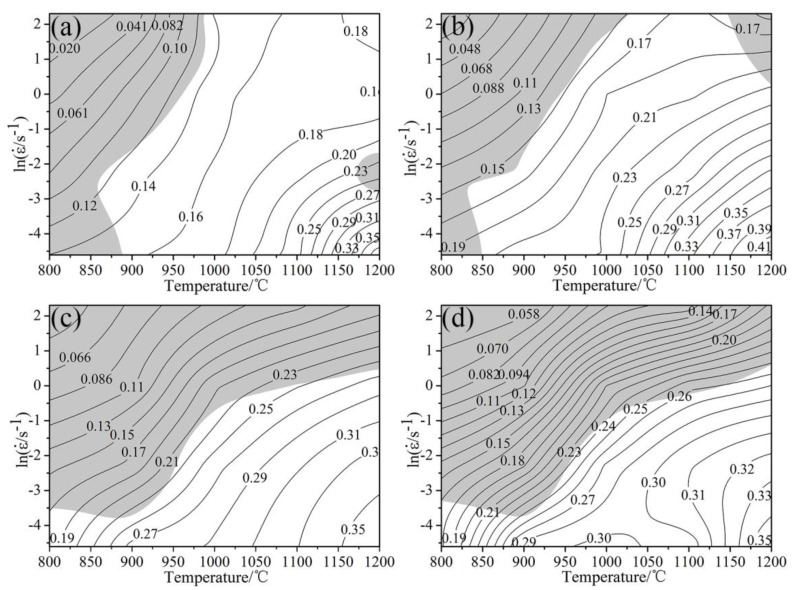
Hot processing maps of the studied steel: (**a**) *ε* = 0.1, (b) *ε* = 0.3, (**c**) *ε* = 0.5 and (**d**) *ε* = 0.7.

Figure 11 exhibits a hot processing diagram of experimental steel at different strains. It can be seen that as the total deformation increased, the instability zone gradually diffused from low temperature and high strain to high temperature and low strain, and the area of the unstable zone gradually increased. In the low strain rate region of about 0.01–0.6 s^−1^ with a deformation temperature of about 1000–1200 °C, it was a safe zone for hot processing. At this time, maximum power dissipation efficiency was obtained in the hot processing map. Thus, it could be assumed to be the best/optimum processing performance for the studied conditions of studied material. Therefore, in the actual production, it is recommended to develop hot working process parameters of Fe-30Mn-0.11C steel in this area.

**Figure 12:**
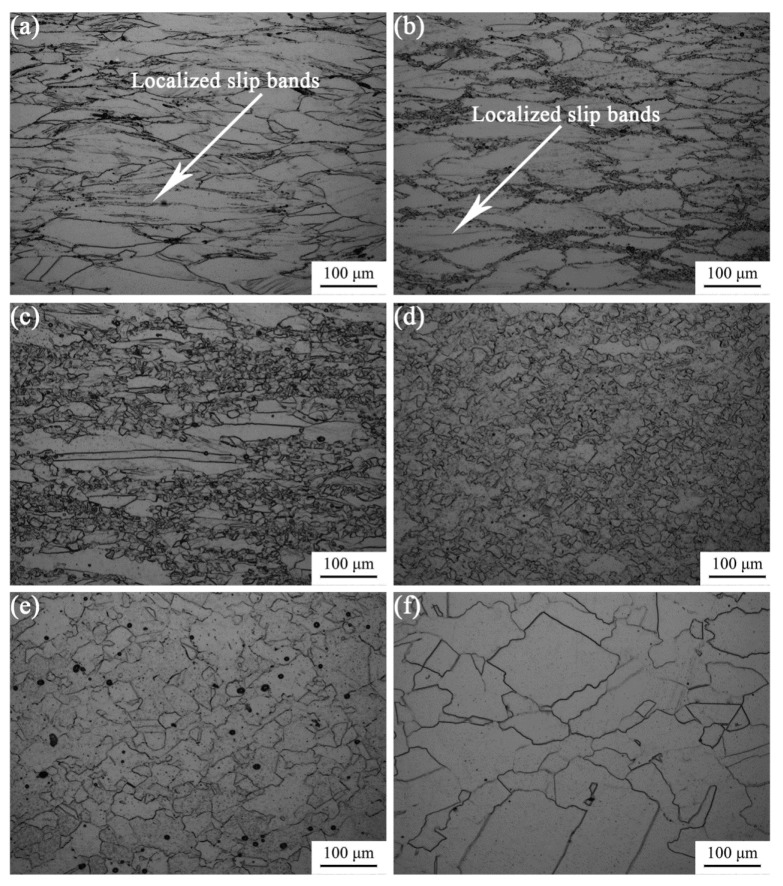
The microstructures of Fe-30Mn-0.11C steel at different deformation conditions: (**a**) 800 °C, 1 s^−1^; (**b**) 900 °C, 1 s^−1^; (**c**) 900 °C, 0.01 s^−1^; (**d**) 1000 °C, 0.01 s^−1^; (**e**) 1100 °C, 0.01 s^−1^ and (**f**) 1200 °C, 0.01 s^−1^.

Figure 12 shows the microstructures of Fe-30Mn-0.11C steel at various deformation conditions. The material is in the thermal processing map at the strain rate of 1 s^−1^ and deformation temperature of 800 and 900 °C. The microstructure displays local adiabatic shear bands in the area, which was one of the rheological instability forms of the material internal structure. Structure changes or heat transfer results in an increase in local temperatures inside the material that caused the appearance of local slips. In addition, less local adiabatic shear bands are found in the sample deformed at 800 °C than that of 900 °C. The hot workability could also be seen in Figure 12c. At a deformation temperature of 900 °C, numerous recrystallized grains are formed between prior austenite grain boundaries. Further, the higher the energy dissipation efficiency is, the more likely the dynamic recrystallization occurs. In high temperature regions where dynamic recrystallization occurred at around 1000–1200 °C, the energy dissipation efficiency was higher and hence, grain growth phenomenon occurred.

It is shown that material possessed the finest dynamic recrystallized grains with a grain size of 10.75 μm at a deformation temperature of 1000 °C and a strain rate of 0.01 s^−1^. It is worth noting that this deformation condition also corresponded to the local peak of the energy dissipation efficiency in the thermal processing diagrams. In addition, this deformation condition was in the safe zone of the thermal processing diagrams at different strains, and has the optimal thermal processing performance. It was further shown that the optimal thermal deformation process parameters of the studied steel were the deformation temperature of 1000 °C and strain rate of 0.01 s^−1^.

## 4. Conclusions

The hot deformation behavior of Fe-30Mn-0.11C steel was systematically studied using hot-compression tests at various temperatures (800–1200 °C) and strain rates (0.01–10 s^−1^). The major results obtained were as follows:

(1) A constitutive equation for the flow stress of the alloy at high temperatures was successfully obtained as a function of deformation temperatures and strain rates. The hyperbolic-sine of the flow stress showed a linear relationship with the Zener–Hollomon parameter.
  $$\dot{\varepsilon }=8.698\times 10^{13}{\left[\sin h(0.01015\cdot \sigma )\right]}^{5.645}\exp(-393,070/RT)$$
(2) The estimated relationships between dynamic recrystallization critical stress (*σ_c_*) with peak stress $\left(\sigma_{p}\right)$ and critical strain ($\varepsilon_{c}$) with peak strain $\left(\varepsilon_{p}\right)$ was obtained to be $\sigma_{c}\;=\;{0.89\sigma }_{p},{\;\varepsilon }_{c}\;=\;{0.4\varepsilon }_{p}$.
(3) The accuracy of the recrystallization size model expressed by the deformation temperatures and strain rates was higher than that of the model represented by the *Z* parameter. The two models established were as follows:
  $$\;D_{DRX}\;=\;{77,365.79Z}^{-0.25}\;$$
  $$D_{DRX}\;=\;{3,592,759\dot{\varepsilon }}^{-0.12763}\exp(\frac{-141,071}{RT})$$
(4) A safe hot working zone locates in the low strain rate zones (about 0.01–0.6 s^−1^) with deformation temperature of 1000–1200 °C, which are the optimal hot deformation process parameters.
(5) Fe-30Mn-0.11C steel possesses good hot workability and the finest grain size at a deformation temperature of 1000 °C and strain rate of 0.01 s^−1^, which are the optimal hot deformation process parameters.
